# Synthesis of Titanium Dioxide Nanoparticles Using *Echinacea purpurea *Herba

**Published:** 2017

**Authors:** Renata Dobrucka

**Affiliations:** *Department of Industrial Products Quality and Ecology, Faculty of Commodity Science, Poznan University of Economics, al. Niepodległości 10, 61-875 Poznan, Poland*

**Keywords:** *Echinacea purpurea*, nanotechnology, green-synthesis, titanium dioxide nanoparticles

## Abstract

Nowadays green synthesis of metal nanoparticles is a developing area of research. In this study, titanium dioxide nanoparticles were biosynthesized using an aqueous solution of* Echinacea purpurea *herba extract as a bioreductant. This is novel and interesting method for synthesis of TiO_2_ nanoparticles. The prepared titanium dioxide nanoparticles were characterized using ultraviolet–visible spectroscopy (UV-VIS), transmission electron microscopy (SEM), total reflection X-Ray fluorescence analysis (TXRF) and Fourier-transform infrared spectroscopy (FTIR)*. *The size of TiO_2 _nanoparticles was found to be in the range of 120 nm. Moreover, the alkaline reaction of the solution (pH = 8) resulted in the increase in absorbance (280 nm), which facilitates the growth of the number of TiO_2 _nanoparticles in the studied solution. Also, synthesis of TiO_2_ nanoparticles using green resources like *Echinacea purpurea *herba is a better alternative to chemical synthesis, since this green synthesis is pollutant-free and eco-friendly.

## Introduction

Nowadays, nanotechnology has been expanding rapidly in recent years, impacting on diverse areas such as the economy and the environment (1). The development of nanotechnology has resulted in a growing public debate on the toxicity and environmental impact of direct and indirect exposures to nanoparticles (2, 3). Moreover, nanoparticles can overcome physiological barriers and readily interact with intracellular compartments without any additional surgery (4). Plant extracts may act both as reducing agents and stabilizing agents in the synthesis of nanoparticles. The source of the plant extract is known to influence the characteristics of the nanoparticles (5). This is because different extracts contain different concentrations and combinations of organic reducing agents (6).

Titanium dioxide (TiO_2_) is an inert, non-toxic and inexpensive material, whose high refractive index and high capability to absorb UV light make it an interesting white pigment and environmentally friendly catalyst (7). The nanosized TiO_2_ particles are widely used to provide whiteness and opacity to products such as sunscreen lotions, paints, plastics, papers, inks, food colorants and toothpastes (8). In literature, TiO_2_ nanoparticles have been synthesized using natural products like *Nyctanthes arbortristis* extract (9), *Catharanthus roseus* (10) aqueous leaf extract, *Eclipta prostrate* aqueous leaf extract (11) and *Annona squamosa* *L*. peel extract (12). So there is a pressing need to develop clean non-toxic and eco-friendly procedures for the synthesis and assembly of nanoparticles (13).

*Echinacea purpurea* is a plant growing in natural in the northern parts of Africa. *E. purpurea* belongs to the family of *Compositae* (*Asteraceae*) and *Asterales*. *Echinacea purpurea* is a medicinal herb commonly known as the purple coneflower, red sunflower and rudbeckia (14). 

*E. purpurea *contains alkamides, cichroic acid and polysaccharides (15). Also,* E. purpurea *is also used to treat chronic infections of respiratory tract and lower urinary tract (viral and bacterial origin). The polysaccharide from *E. purpurea* used to kill bacteria such as staphylococci. *Echinacea purpurea *has potent to activate macrophage cytotoxicity actions against tumor cells and micro organisms (16). In this study, TiO_2_ nanoparticles were synthesized using *Echinacea purpurea *herba by simple aqueous reduction method.

## Experimental

*Preparation of TiO*_2_ *nanoparticles*

**Figure 1:**
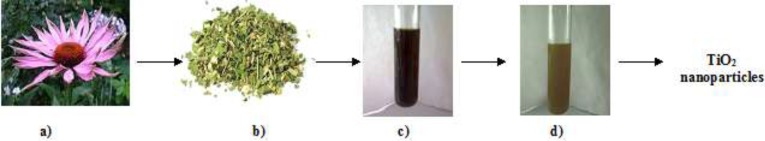
Schematic illustration of the green synthesis of TiO_2_ nanoparticles using aqueous extract of the *E. purpurea *herba, a)* E. purpurea *plant, b)* E. purpurea *powder, c) plant extract solution, d) plant extract with 1mM TiO_2_

Aqueous extract of *E. purpurea* was prepared using 10 g herba boiled with 50 mL of double distilled water at 90 °C for 20 min. This extract was filtered through a medium filter. 1 mM TiO_2_ (aq) solution was stirred for 2 h in 25 °C to prepare nanoparticles of TiO_2_. 10 mL of the aqueous extract of *E. purpurea* were added to 20 mL of 1 mM TiO_2_ at 25 °C, under stirring condition for 4 h. After 4 h, the color of the extract with TiO_2 _nanoparticles changed to green. Figure 1 shows the schematic illustration of the green synthesis of TiO_2_ nanoparticles using aqueous extract of the *E. purpurea *herba.

*Characterization of TiO*_2_ *nanoparticles*

The UV–visible spectroscopy is a commonly used technique (17) in the characterization of nanoparticles. Light wavelengths in the 300–800 nm are generally used for characterizing various metal nanoparticles in the size range of 2 to 100 nm (18) and more. Also, the optical property of TiO_2_ nanoparticles was analyzed via ultraviolet and visible absorption spectroscopy (spectrophotometer Cary E 500) in the range 200 -400 nm. 

The morphology of the plant-synthesized TiO_2_ nanoparticles was examined by means of scanning electron microscopy (SU3500, Hitachi with spectral imaging system Thermo Scientific NSS (EDS), the tape of detector (BSE-3D), acceleration voltage (15.0 kV), working distance (11.6 mm), pressure (40 Pa). The characterization involved Fourier transform infrared spectroscopy (FTIR) analysis of the dried powder of synthesized TiO_2_ nanoparticles by Perkin Elmer Spectrum 1000 spectrum, in attenuated total reflection mode, and using the spectral range of 4000–400 cm−1, with a resolution of 4 cm−1. The presence of TiO_2 _nanoparticles in the extract of *E. purpura* was confirmed using X-ray fluorescence spectrometer Bruker S2 TXRF Picofox, operated at 50 KV and 600 uA.

## Results and Discussion

*UV-VIS spectra analysis*

**Figure 2:**
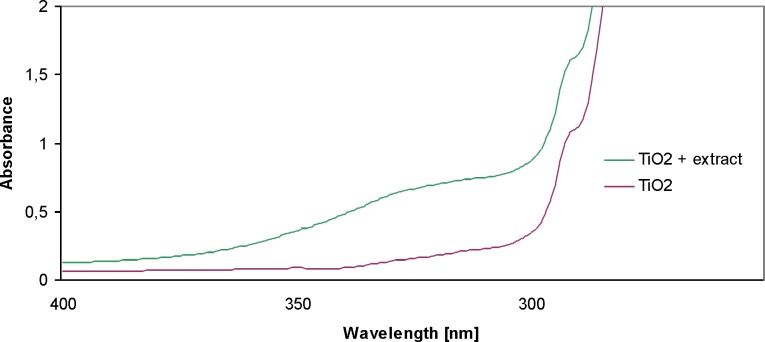
UV-Vis absorption spectra of titanium nanoparticles synthesized using E. purpurea herba extract

In order to confirm the presence of nanoparticles in the resulting solutions, the UV-Vis spectra were analyzed. UV-Vis absorption spectroscopy is an important technique to monitor the formation and stability of metal NPs in aqueous solution. The absorption spectrum of metal NPs is sensitive to several factors, including particle size, shape, and particle–particle interaction (agglomeration) with the medium (19). The absorption maximum (λmax) depends on the nanoparticles, the size and shape (20). Figure 2 shows the UV-VIS absorption spectra for TiO_2_ nanoparticles between 200 and 400 nm. The absorption for TiO_2_ appears at 280 nm in UV–Vis spectroscopy. Similar studies were conducted by Roopan (12). On the spectrum, the sample 1 nM TiO_2_ and the sample of 1 nM TiO_2_ + extract were analyzed. The particle suspension was diluted to 1:100 with distilled water, to avoid errors due to high optical density of the solution. The spectrum of TiO_2_ showed a marked increase in the absorbance of the solution of TiO_2_ + extract.

**Figure 3:**
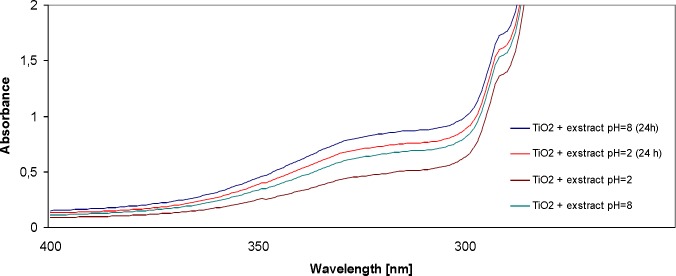
UV-Vis absorption spectra of titanium nanoparticles (pH = 2 and pH = 8 after preparation and after 24 h) synthesized using E. purpurea herba extract

In the next stage, two solutions were prepared. All assays were performed in a dilution of 1: 100. The pH of one solution was 2 (acidic), and of the other – 8 (alkaline). The aim of the study was to determine the influence of the reaction of the solution on the formation of TiO_2_ nanoparticles. The conduced studies made it possible to determine that the alkaline reaction of the solution (pH = 8) led to the increase in absorbance. The peak was observed at 280 nm. The increase in absorbance was recorded for the solution after preparation (4 h of stirring), as well as after 24 h, with the constant pH = 8. As regards the solution with pH = 2, changes in absorbance were observed 4 and 24 h after preparation; however, the values were lower than in the case of alkaline solutions. The increase in absorbance may reflect the growing number of TiO_2_ nanoparticles in the examined solution. Figure 3 shows the spectrum of the TiO_2_ + extract after changing the pH to 2 and 8, immediately after preparation, as well as the spectrum of the same solutions after 24 h. 

**Figure 4:**
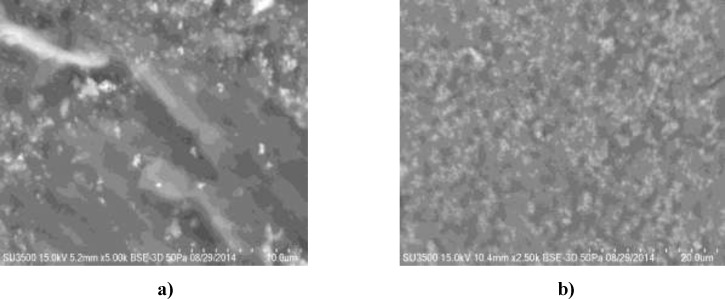
Scaning electron microscopy of titanium nanoparticles synthesized using E. purpurea herba extract. The scale bar: a) 10 μM, b) 20 μM

**Figure 5:**
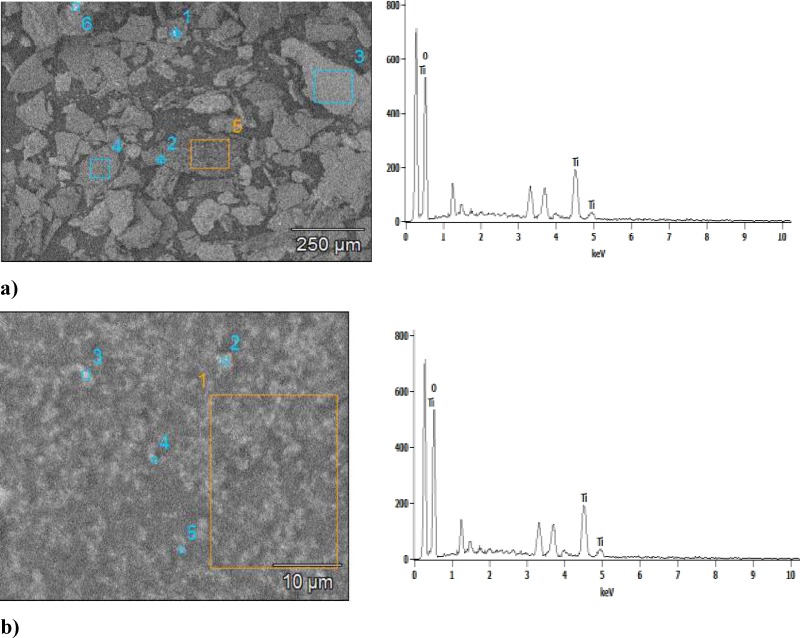
Scaning electron microscopy of titanium nanoparticles synthesized using E. purpurea extract and EDS profile, a) Image Pixel Size: 2.51 µM , b) Image Pixel Size: 0.10 µM

**Figure 6:**
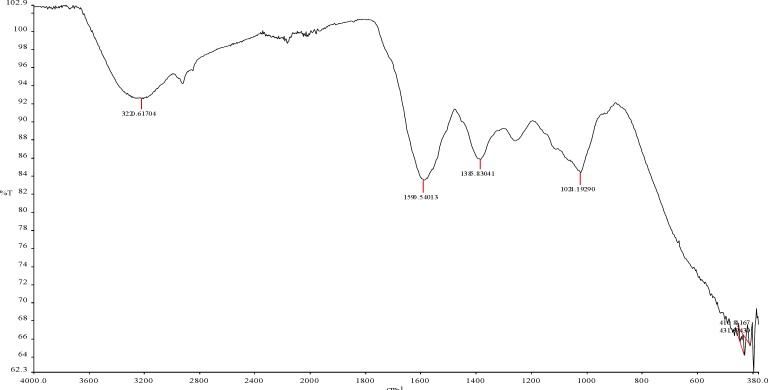
FTIR spectra of titanium nanoparticles synthesized using E. purpurea herba extract

**Figure 7:**
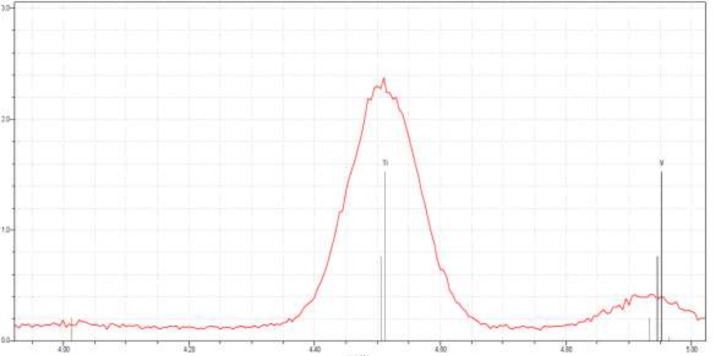
TXRF spectrum of titanium nanoparticles synthesized using E. purpurea herba extract

*Scanning electron microscopy (SEM)*

The use of scanning electron microscopy (SEM) made it possible to provide information on the size of TiO_2_ nanoparticles. The plant-synthesized TiO_2_ nanoparticles are quite polydisperse and they range in size about 120 nm (Figure 4a and 4b. The scale bar is 10 μM and 20 μM). The numbers 1, 2, 3, 4, 5 and 6 indicate the points in which the measurement was made. To gain a further insight into the features of TiO_2 _nanoparticles, the analysis of the sample was performed using EDS techniques. The nanoparticles are poorly dispersed, with spherical clusters with agglomeration. Agglomeration makes it difficult to study individual nanoparticles (21). In addition, the presence of TiO_2_ nanoparticles was confirmed in the sample. Quantification was studied for each of the areas. This confirmed the presence of TiO_2_ nanoparticles in the sample. Also, Figure 5 presents SEM of TiO_2 _nanoparticles synthesized using *E. purpurea *extract and EDS profile with image pixel size: 2.51 µM and image pixel size: 0.10 µM.

*Fourier transform infra-red spectroscopy (FTIR)*

FTIR spectroscopy was used to determine different groups on *E. purpurea* powder and predict their role in nanoparticle synthesis. In Figure 6 it is observed that the bands are at 3320 cm-1, 1590 cm-1, 1385 cm-1, 1024 cm-1. and 431 cm-1. The FTIR spectrum of TiO_2_ nanoparticles showed characteristic bands at 1024 cm-1 that indicate the presence of C-O stretching alcohols, carboxylic acids, esters and ethers. The peak at 1385 cm-1 indicates C-H rock alkenes, while the peak at 1590 cm-1 indicates the presence of C=C characteristic of saturated hydrocarbons. The band at 3320 cm-1 corresponds to O-H, as also the H-bonded alcohols and phenols. These bonds are related to the chemical composition of *E. purpura*. Also, *E. purpurea* composition includes such compounds as caffeic acid, quinic acid, and chlorogenic acid. Furthermore, it includes flavonoids, in the free form and glycosidically bound (quercetin, kaempferol, rutoside, luteolin, apigenin, isorhamnetin), and essential oil. The peak at 431 cm-1 indicates the presence of TiO_2_ nanoparticles.

*Total Reflection X-Ray Fluorescence Analysis (TXRF)*

The elemental composition of the green-synthesized sample was also studied using X-ray fluorescence spectrometer TXRF Bruker S2 Picofox, operated at 50 KV and 600 μA. 

Total reflection X-ray fluorescence (TXRF) is a comparatively multielement analytical technique for trace element determinations (22). TXRF method is based on the fact that each element contained in the sample, as a result of X-ray excitation, emits its characteristic spectrum, based on qualitative and quantitative analysis. The TXRF spectrum, shown in Figure 7, reveals the clear elemental composition profile of the green-synthesized titanium nanoparticles. The intense signal at 4,5 KeV strongly suggests that titanium nanoparticles were the major element of *E. purpurea *herba extract. 

## Conclusion

The present study shows the environmentally benign, low cost, and renewable approach for the synthesis of TiO_2_ nanoparticles using *Echinacea purpurea *herba extract as reducing agent. The presence of TiO_2_ nanoparticles was confirmed by means of UV–VIS spectroscopy, FTIR spectroscopy and TXRF. The size of the nanoparticles was measured using SEM-EDS. The average size of the obtained nanoparticles was around 120 nm. Therefore, this method is very effective in order to obtain TiO_2 _nanoparticles. It should be added that this method is ecological, as well as quick and it uses the temperature below 100 °C. In the current literature, we encounter numerous examples of obtaining TiO_2 _nanoparticles using numerous chemical substances and high temperature, often exceeding 400 °C. Moreover, the influence of solution pH on the formation of nanoparticles was established. The alkaline reaction of the solution (pH = 8) resulted in the increase in absorbance (280 nm), which facilitates the growth of the number of TiO_2 _nanoparticles in the studied solution.

## References

[1] Ahmed M, Karns M, Goodson M, Rowe J, Hussain SM, Schlager JJ, Hong Y (2008). DNA damage response to different surface chemistry of silver nanoparticles in mammalian cells. Toxicol. Appl. Pharmacol..

[2] Brayner R (2008). The toxicological impact of nanoparticles. Nanotoday.

[3] Panda KK, Achary VMM, Krishnaveni R, Padhi BK, Sarangi SN, Sahu SN (2011). In vitro biosynthesis and genotoxicity bioassay of silver nanoparticles using plants. Toxicol. In Vitro.

[4] Amjadi I, Rabiee M, Hosseini MS (2013). Anticancer activity of nanoparticles based on PLGA and its Co-polymer: In-vitro Evaluation. Iran. J. Pharm. Res..

[5] Kumar V, Yadav SK (2009). Plant-mediated synthesis of silver and gold nanoparticles and their applications. J. Chem. Technol. Biotechnol..

[6] Mukunthan K, Balaji S (2012). Cashew apple juice (Anacardium occidentale L) speeds upthe synthesis of silver nanoparticles. Int. J. Green Nanotechnol..

[7] Zhao J, Yang X (2003). Photocatalytic oxidation for indoor air purification: a literature review. Build. Environ..

[8] Trouiller B, Reliene R, Westbrook A, Solaimani P, Schiestl RH (2009). Titanium dioxide nanoparticles induce DNA damage and genetic instability in vivo in Mice. Cancer Res..

[9] Sundrarajan M, Gowri S (2011). Green synthesis of titanium dioxide nanoparticles by Nyctanthes arbor-tristis leaves extract. Chalcogenide Lett..

[10] Velayutham K, Rahuman AA, Rajakumar G, Santhoshkumar T, Marimuthu S, Jayaseelan C, Bagavan A, Kirthi AV, Kamaraj C, Zahir AA, Elango G (2011). Parasitol. Res.

[11] Kirthi AV, Rahuman AA, Rajakumar G, Marimuthu S, Santhoshkumar T, Jayaseelan C, Elango G, Zahir AA, Kamaraj C, Bagavan A (2011). Mater. Lett..

[12] Roopan S M, Bharathi A, Prabhakarn A, Rahuman AA, Velayutham K, Rajakumar G, Padmaja RD, Leksami M, Madhumitha G (2012). Efficient phyto-synthesis and structural characterization of rutile TiO2 nanoparticles using Annona squamosa peel extract. Spectrochim Acta Part A.

[13] Mukunthan K, Balaji S (2012). Cashew apple juice (Anacardium occidentale L) speeds up the synthesis of silver nanoparticles. Int. J. Green Nanotechnol..

[14] Kumar KM, Ramaiah S (2011). Harmacological Importance of Echinacea Purpurea. Int. J. Pharma Bio Sci..

[15] Li T (1998). Growing Echinacea.

[16] Pullaiah T (2006). Encyclopedia of world medicinal plants.

[17] Pal S, Tak YK, Song JM (2007). Does the antibacterial activity of silver nanoparticles depend on the shape of the nanoparticle? A study of the gram-negative bacterium Escherichia coli. Appl. Environ. Microbiol..

[18] Feldheim DL, Foss Jr CA (2002). Metal Nanoparticles; Synthesis, Characterization and Applications.

[19] Aryal S, Bahadur KCR, Bhattard N, Kim CK, Kim HY (2006). Study of electrolyte induced aggregation of gold nanoparticles capped by amino acids. J. Colloid Interface Sci..

[20] Sönnichsen C, Franzl T, Wilk T, von Plessen G, Feldmann J, Wilson O, Mulvaney (2002). Drastic reduction of plasmon damping in gold nanorods. P. Phys. Rev. Lett..

[21] Rajakumar G, Abdul Rahuman A, Priyamvada B, Gopiesh Khanna V, Kishore Kumar D, Suzin PJ (2012). Eclipta prostrata leaf aqueous extract mediated synthesis of titanium dioxide nanoparticles. Materials Lett..

[22] Misra NL (2011). Total reflection X-ray fluorescence and energy-dispersive X-ray fluorescence characterizations of nuclear materials, Pramana. J. Phys.

